# Core Legal Challenges for Medical 3D Printing in the EU

**DOI:** 10.3390/healthcare12111114

**Published:** 2024-05-29

**Authors:** Ante B. V. Pettersson, Rosa Maria Ballardini, Marc Mimler, Phoebe Li, Mika Salmi, Timo Minssen, Ian Gibson, Antti Mäkitie

**Affiliations:** 1 Department of Otorhinolaryngology-Head and Neck Surgery, University of Helsinki and Helsinki University Hospital, FI-00029 HUS, Helsinki, Finland; 2 Department of Vascular Surgery, University of Helsinki and Helsinki University Hospital, 00100 Helsinki, Finland; 3 Faculty of Law, University of Lapland, 96399 Rovaniemi, Finland; rosa.ballardini@ulapland.fi; 4 The City Law School, City, University of London, London EC1V 0HB, UK; marc.mimler@city.ac.uk; 5 Sussex Law School, University of Sussex, Brighton BN1 9RH, UK; phoebe.li@sussex.ac.uk; 6 Department of Mechanical Engineering, Aalto University, 02150 Espoo, Finland; mika.salmi@aalto.fi; 7 Center for Advanced Studies in Bioscience Innovation Law (CeBIL), Faculty of Law, University of Copenhagen, 1172 Copenhagen, Denmark; timo.minssen@jur.ku.dk; 8 Department of Design, Production and Management, University of Twente, 7522 NB Enschede, The Netherlands; i.gibson@utwente.nl; 9 Research Program in Systems Oncology, Faculty of Medicine, University of Helsinki, FI-00014 Helsingin yliopisto, Helsinki, Finland

**Keywords:** additive manufacturing, 3D printing, legal issues, legislation, regulation, medical, medicine

## Abstract

3D printing has been adopted into routine use for certain medical applications, but more widespread usage has been hindered by, among other things, unclear legislation. We performed an analysis, using legal doctrinal study and legal informatics, of relevant EU legislation and case law in four issues relevant to medical 3D printing (excluding bioprinting or pharmacoprinting): pre-market approval, post-market liability, intellectual property rights, and data protection. Several gaps and uncertainties in the current legislation and interpretations were identified. In particular, we regard the current EU regulatory framework to be quite limiting and inflexible, exemplifying a cautionary approach common in EU law. Though the need to establish high safety standards in order to protect patients as a disadvantaged population is understood, both legal uncertainties and overregulation are seen as harmful to innovation. Hence, more adaptive legislation is called for to ensure continuous innovation efforts and enhanced patient outcomes.

## 1. Introduction

### 1.1. Medical 3D Printing

3D printing (3DP) or additive manufacturing (AM) has, in the last few years, reached a stage of maturity where it has been adopted by several industries. In 2017, the healthcare sector represented ca 11% of the AM market [1]. 3DP has two main benefits compared to traditional manufacturing technologies. First, it enables the manufacturing of geometries, surfaces, and internal structures that would either be expensive or impossible to produce by other means and at no extra cost. This benefit of 3DP is currently utilized in the production of several mass-produced medical devices, such as orthopedic implants. Second, it enables the production of short runs or even single objects, and together with advanced Computer-Aided Design (CAD) software and parametric design, it enables the easy production of customized products. This has led to a growing number of medical devices tailored to the patient’s unique requirements and anatomy, variably known as patient-specific, patient-matched, customized, or custom devices. For a current classification of AM technologies and terminology, see [2].

From a regulatory perspective, mass-produced 3DP is in many ways (e.g., chain of production) like traditional manufacturing, the only exceptions being certain technical properties that are unique to the process, such as anisotropy (different mechanical properties depending on dimension).

Patient-specific products on the other hand have several unique legal issues and will serve as the basis for the majority of discussion in this article. Besides traditional medical device manufacturers, these devices may in some cases also be manufactured on a 3D printer located at a healthcare facility. It may be worth noting that even though 3DP is seeing the fastest growth, some of these patient-specific products may be produced by other Computer-Aided Manufacturing (CAM) methods such as milling, or even manually.

Patient-specific medical 3DP products can be divided into medical models, implants, tools, external aids, and biomanufacturing, the last of which is not yet in clinical use. Examples currently in use include preoperative planning models, tools such as saw guides, and personalized cranial implants which are in wide use in reconstructive operations concerning the head region. External aids are currently best exemplified by the personalized retainers that have become common in dentistry.

### 1.2. The Medical 3D Printing Process

**Figure 1:**
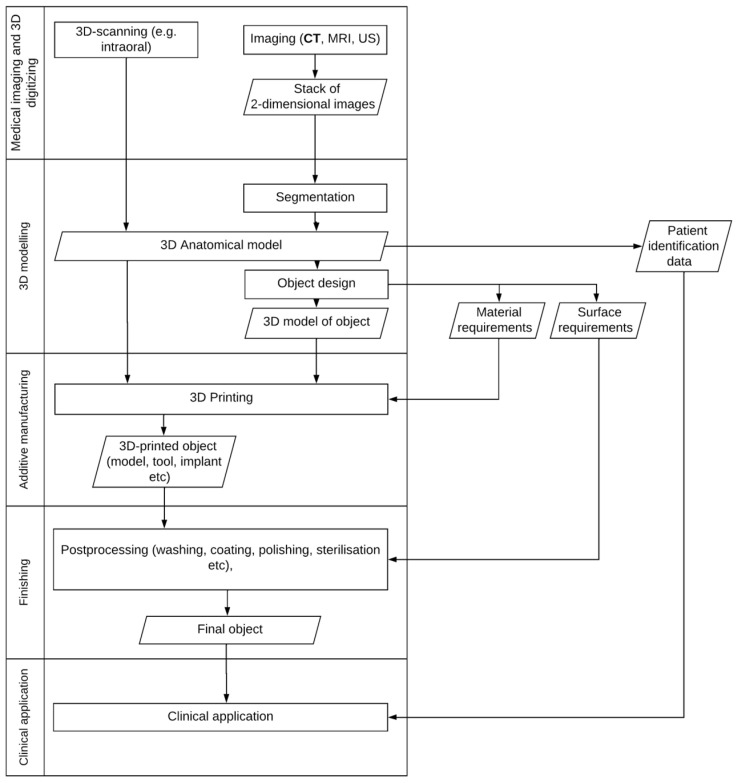
A process description of clinical 3D printing. Physical objects, documents, software, and data are represented by parallelograms, while various steps of the process are described by rectangles. Each of these steps contributes to the final quality of the product. A 5-step classification of the various processes is presented on the left.

To understand the legal issues concerning medical 3DP, one must first understand the process. While we here discuss 3D printing specifically, many of these steps apply to any patient-matched medical device regardless of the manufacturing methodology (Figure 1).

As seen in the figure, the 3DP process facilitates a distributed manufacturing chain. In some cases, the designer may also be the surgeon, and the print is produced in the hospital, whereas in others, the manufacturing or even the design may be outsourced to a service provider, which in turn sometimes further sources certain types of printing (such as metallic AM) from subcontractors. This may have legal ramifications, as discussed later.

#### 1.2.1. Medical Imaging or 3D Digitizing

Imaging data are typically acquired by the radiology department which has its own quality control process to ensure that imaging devices are correctly calibrated etc. Most often computed tomography (CT) imaging is used, but magnetic resonance imaging (MRI) and ultrasound (US) are other alternatives. The result is typically a series of grayscale 2D images saved together in a DICOM (Digital Imaging and Communications in Medicine) file, containing patient identifying information. Imaging for 3D printing has certain unique requirements that should be considered from the very beginning to produce an optimal model.

#### 1.2.2. 3D Modeling

In the second step, the 2D images are converted into a triangulated 3D surface model, producing a 3D file format such as STL. This process, called segmentation, requires special software and user input to determine which grayscale values are considered part of the object and which are background, as well as possibly applying various manipulations to get rid of noise and artifacts. In certain cases, especially in dentistry, a 3D scanner may be used to directly digitize anatomy into a 3D model in the first phase, obliterating the need for separate segmentation.

If the planned product is something other than an anatomical model, the next step is to use the 3D surface model to design the final product using various CAD software. Besides a standard engineering approach, it is possible to use automated methods such as finite-element analysis and parametric design to optimize the design or to adapt a single design to multiple patients. The result of the design phase is also a 3D surface model.

#### 1.2.3. Additive Manufacturing

In the third step, the 3D surface models are converted into layers and commands (G-code) for controlling the printer and finally printed to produce physical objects. Designers may in the previous step provide specifications or outright choose the material which the printer operator will use. When structural integrity is required, several replicas may be printed for destructive testing.

#### 1.2.4. Finishing

In many cases, especially outside of medical models, the printed product is not directly suited for clinical use. The penultimate step includes post-processing such as washing to remove supports or unsolidified raw materials, polishing and other surface treatments, and sterilization. Some of these steps (like washing and support removal) may be performed by the printer operator, while others (such as sterilization) may be performed by the end user. Requirements for surface quality, etc., may be decided by the designer.

#### 1.2.5. Clinical Application

The final step is the clinical application of the product. Typically, the clinician who ordered the print will also be the one using it. Lastly, in the case of patient-specific implants, postoperative imaging can be used to close the loop and evaluate the spatial accuracy of the whole process.

## 2. Materials and Methods

In a previous paper we performed a scoping review of publications in legal, medical, and technical journals concerned with regulation and legal issued of medical 3DP [3]. Based on this study, we identified patient-specific products (rather than off-the-shelf products produced with 3DP) as the crux of the legal issues surrounding the methodology, and specifically identified the problems to occur in the legal fields of pre-market regulation, liability, intellectual property, and privacy/data protection.

The legal scholars comprising part of the research team were then tasked with exploring these topics further. While this is a topic with global relevance, law is territorial, and based on our location (i.e., Europe) we chose to focus on the European legal framework.

We implemented methodologies traditionally used in legal analysis, namely legal doctrinal analysis and normative approaches, combined with legal informatic (LI) methodologies. The legal doctrinal method was used to map the relevant legal and underlying policy framework as well as the legal practices applicable to medical 3DP. The legal doctrinal method produces information about the law (i.e., it describes the law) and systematizes the legal norms [4]. The usual aim of this type of description is “to present the law in a certain field” (e.g., in data protection and privacy law) in a way that is “as neutral and consistent as possible, in order to inform the reader how it actually reads” [5]. In other words, the doctrinal method in law aims to show how the law should be interpreted. Thus, we allocated existing primary sources of law, i.e., statutory and case law, in particular those produced by the EU legislature and courts, and described them as they apply to medical 3D printing. Our aim was to make the legal sources easier to understand also by a non-legal audience—the importance of which was underscored by the multidisciplinary nature of both the topic under investigation and the research team itself. 

Moreover, to explore the current opportunities and barriers created by EU rules on IPR, data protection, and liability, as well as suggest more suitable future paths, we applied problem-solving methodologies from legal informatics (LI). LI is a discipline “familiar with future scenario analysis and aimed at exploiting technology to the maximum extent possible, while minimizing the legal, ethical, social and economic risks” [6]. The methodology used in LI is based on a mixed multidisciplinary, international, and comparative approach. In this case, LI provided a common holistic approach to the technical and legal issues related to medical 3DP. As such, both the legal doctrinal and normative approaches and the LI methods were crucial to provide normative de lege ferenda recommendations for future legislative and policy developments on how the law should be in this context.

## 3. Results

### 3.1. Regulation and Liability

The regulatory regime for medical devices is divided into two phases: pre-market (ex-ante) and post-market (ex-post). While the former concerns the regulatory safety and market approval pathway, the latter involves additional regulatory obligations, as well as liability issues arising from product defects or negligence after being authorized and licensed for use (Table 1).

#### 3.1.1. Pre-Market (Ex-Ante)

Pre-market regulation of medical devices in the EU is based on a risk classification similar, though not identical to, that of several other jurisdictions. Band-aids would fall into the lowest risk class (Class I) and require the least amount of testing before entering the market, while at the other end of the spectrum life-supporting products would end up in Class III and require extensive evidence of safety. The method of production itself does not influence the risk class, so 3DP products may be found in any risk class [7].

Previously governed by the Medical Device Directive, the new EU Medical Device Regulation (MDR) of 2017 [8] is currently in effect since 26 May 2021. Even before coming into effect, the MDR was criticized for not directly addressing 3DP or personalized medical devices [9], and a separate document regarding personalized devices was later released to answer some of the questions left open by the MDR [7].

One of the key questions relates to product classification and the market authorization pathway under the Medical Device Regulation (MDR). In other words, does a device require market authorization or certification before entering the market?

Although some earlier approaches considered regulating 3D printers themselves as medical devices, this idea has since been mostly abandoned. The software used in the 3DP toolchain may, however, under the new MDR fall under the definition of “Software as a medical device”. If the intended purposes and functions of software used for 3D printing affect medical decision-making, it is qualified as a medical device under the MDR and is subject to safety and performance requirements.

A big question affecting medical 3DP is the regulation of ‘mass customized’ devices under the new MDR. The MDR has a Custom Device Exemption that exempts devices “made in accordance to a written description… [by a] person authorized by national law...under that person’s responsibility” from certain requirements of the MDR, such as obtaining CE-marking (Article 52.8 and Annex XIII). The Custom Device Exemption, however, does not exempt a product from most requirements, such as having a quality management system, etc. It is noteworthy that the MDR makes a distinction between customized devices and industrial mass-produced devices needing adaptation following prescription (Article 2.3). Devices would not be considered customized if printed following a specific range of pre-determined measurements which require individual adaptation after printing [10]. The scope of the Custom Device Exemption is one of the more open questions left in the MDR, and later guidance has partly aimed at settling some of these questions [7], specifically saying that “patient-matched devices […] matched to a patient’s anatomy within a specified design envelope […] produced through a process that is capable of being validated and reproduced” do not fulfill the requirements of the Custom Device Exemption and must follow the more stringent regulatory pathway.

Unlike the US regulatory landscape, where the FDA is concerned mostly with interstate trade but has limited powers to influence what happens inside hospitals [11], the EU MDR regulates both devices that are “placed on the market” as well as those manufactured and used within health institutions, which are viewed as “put into service” (Article 5). The MDR, however, provides a Health Institution Exemption by which in-house non-industrial manufacturing of 3DP devices would be exempt from certification and certain other requirements, although still subject to, among other things, having a quality management system (Recital 30 and Article 5.5).

#### 3.1.2. Post-Market (Ex-Post)

In addition to the general post-market requirements on surveillance and monitoring, product liability is another key theme after a product is licensed and enters the market or service. The interplay between pre-market regulations and post-market liability is complex. Internationally, under some jurisdictions (notably the US), compliance with pre-market regulations may act as a full defense for the manufacturer from potential liability claims [12], but in the EU it provides only a partial defense [13,14].

Liability claims arising in a medical 3DP setting can be divided into various categories, including clinical negligence or malpractice claims and product liability. Following the standard practice in the field, surgeons bear the duty of care for their patients and are expected to examine and review the quality of a 3DP device before implantation into the human body. Failing to do so would be grounds for medical negligence claims.

Product liability is distinct from liability for medical malpractice. The product liability regime for 3DP technologies becomes highly relevant particularly after the technology has been licensed or authorized for use [10]. In the EU, the 1985 Product Liability Directive [15] is the main instrument setting out relevant rules on product liability. Currently, the EU is undergoing a major revision of the product liability regime, which we will expand on below.

Product liability focuses on ‘defects’ of a product, which may arise from any process in the chain of custody. Typical adverse events for a medical device include incompatibility and device fracture. A range of defects in 3DP products that would possibly lead to product liability claims include defective imaging, defective original digital designs, defective digital files, defective printers, and defective materials. Human error would contribute to a defect; however, this falls under the negligence regime.

The decentralized production model of 3DP complicates risk management throughout the chain of custody. Attribution of liability will vary depending on whether 3DP is performed in-house or by external third-party providers. It may be hard to tell who the ‘manufacturer’ of a product is, and what exactly is the ‘final product’ at issue, two terms which are crucial for traditional liability law. The demarcation of actors’ liability, therefore, becomes the crux of the post-market traceability requirement.

In short, key questions for liability in a 3DP context are as follows:

1. What are the attributable liabilities;
2. The demarcation of liability between different actors;
3. The definition of a ‘defect’ in a product;
4. Determining who the ‘manufacturer’ is.

Currently, the recently proposed EU Product Liability Directive is at the center of debates [16]. Among other things, it aims to broaden the scope of the previous Directive to better reflect the defects and compensations incurred in the digital age. The proposed revision of the definition of ‘product’ includes intangible goods such as software and digital files, digital services, connectivity, and data. The definition of ‘damage’ is proposed to be broadened by including material loss from corruption of data.

The proposed reform also broadens the scope of liable parties compared to the existing PLD. Any ‘economic operator’ who has substantially modified the product outside the control of the manufacturer will be considered a manufacturer. A layered approach is mapped out to regulating different types of ‘economic operators’: (1) the manufacturer of a product or component, (2) the service provider, (3) the authorized representative, (4) the importer, and (5) the distributor or the fulfillment service provider [16].

Product liability claims would not necessarily single out the detailed process but overall refer to the integrity of the final product [17]. Under the existing PLD, the one who delivered the ‘final’ product is normally responsible for the product defect, so the finishing lab bears the duty of care for the integrity of the final product. However, if the product is then passed on to the surgeon, the surgeon would also bear the duty of care for inspection before implementation. A general demarcation line is that the surgeon is liable for medical malpractice, while the medical device company (if third-party provision) bears the product liability. However, the boundary between medical negligence and product liability is blurred in the case of in-house 3DP, as it is unclear whether the finishing lab or the surgeon delivers the final product. Under the proposed new PLD, any actors that have substantially modified the digital file, software, and/or data would be liable as a manufacturer. Therefore, it is likely that the service providers, such as health institutions and surgeons, as well as device providers would be jointly liable for defects.

Notably, the standard of safety is not an absolute concept but a relative one and depends on the general public’s expectations. It is established in the EU/UK that it is unrealistic to expect 100% safety of a medical product, and that the appropriate standards of safety are dependent on factors including advertisement claims and instructions or warnings attached to the product (Wilkes v DePuy International Limited, paras 13, 22, 102) [14]. Further, the ‘Learned Intermediary Doctrine’ provides an exception to the general rule of product liability by discharging manufacturers’ liability with healthcare practitioners’ duty to warn the patients of possible risks. It is argued that a product is not defective if passing the warning to a physician. This doctrine in general encourages practitioners’ duty to engage in the informed consent process with their patients. After all, a doctor’s advice would normally be of more credence than the producer’s warning [13,14]. The duty of physicians to disclose risks of treatment was also discussed in the UK in Montgomery v Lanarkshire Health Board [18], where it was ruled that physicians are under a duty to take reasonable care to ensure that their patients are “aware of any material risks involved in any recommended treatment, and of any reasonable alternative or variant treatments”. The Court stressed the importance of ‘dialogue’ on the benefits of the treatment, the alternatives available, and the risks involved.

In EU case law, in the case of Boston Scientific [19], the Court of Justice of the European Union (CJEU) ruled that the concept of “damage caused by death or personal injuries” used in the PLD must be given “a broad interpretation”, in this case to include the need for surgical replacement of a medical device classified as defective. Additionally, recognizing the safety requirement for medical devices to be particularly high, it ruled that all medical devices “belonging to the same group or forming part of the same production series” may be classified as defective if a potential defect is found in the group. This interpretation may suggest that all 3DP devices produced by the same process will be susceptible to classification as defective if one defect is identified in an equivalent product.

In comparison, the English Court held a more stringent approach to determining when a product is defective in Wilkes v DePuy [14]. Even though it had been determined that an artificial joint in the patient’s body had fractured and the design of the device made it susceptible to such a failure, the device was ruled not to be defective as the failure was rare but recognized, the design had other desirable characteristics, it had received pre-market approval, and sufficient warning had been provided. According to this ruling, determination of a defect requires consideration of all relevant circumstances, to include risk–benefit balance, avoidability of the defect, whether the product was within the manufacturer’s specification, compliance with standards and regulations, provided warnings and instructions for use, and the involvement of a professional healthcare intermediary.

It is noteworthy that there are legitimate defenses for product liability of 3D printed medical devices in the EU Product Liability Directive. A ‘development risks defense’ allows a defense where the risks of a product could not be identified with the state of scientific and technical knowledge at the time. The CJEU has taken the position that once an isolated opinion suggests there might be a risk, and this information is accessible, the risk cannot be said to have been unforeseeable [20]. In the new proposed PLD, the ‘development risk defense’ would no longer be applicable [16].

As mentioned above, another partial defense is related to ex-ante regulatory compliance. While regulatory compliance may not be a full defense, it may provide strong evidence of the relative level of safety of the product [10].

### 3.2. Intellectual Property Rights

#### 3.2.1. IPR Basics

Various areas of intellectual property (IP) law are relevant with regard to the steps of the medical 3DP process described in Figure 1. Before delving into specifics, some basic information on IP law and IP rights (IPR) is necessary.

IP laws aim at incentivizing innovative and creative activities by awarding an exclusive, temporary, and limited right to the creator of an artistic work or the inventor of a technical innovation, while also balancing societal interests [21].

IPR comprises various types of rights, the most relevant for 3DP being copyright, patent, trademark, and design rights. In simplified terms, copyright protection attaches to original works of intellectual labor, while patents may be granted for technical inventions that are new, involve an inventive step, and are capable of industrial applicability (and are sufficiently disclosed). Trademarks may be registered for distinctive signs enabling the separation of one trader’s goods and services from those of others. Design rights (registered) protect the visual appearance of a product or item. IP rights entail the right to exclude others, or to deny others, from the use of the subject matter in question: copyright signifies exclusive rights to authors with regard to, e.g., reproducing and disseminating their works, patent protects inventions from being used, sold, imported, etc., by third parties without authorization, and a protected sign is reserved for the trademark holder’s use in the course of trade and designs and provides an exclusive right to stop unauthorized parties from producing or using the design [22] (see also Table 2).

Focusing on the system of IPR in the European Union, several attempts have been made over the years to harmonize the different areas of European IP law, and the result is quite a large amount of EU Directives and Regulations, as well as several jurisprudential interpretations by the Court of Justice of the European Union (CJEU). Nevertheless, national law and case law are also present and relevant, with some areas of IPR still holding quite a national-based touch.

#### 3.2.2. IPRs and 3DP

The steps of the medical 3DP process that raise the most challenges concerning IPR are 3D modeling (step 2) and additive manufacturing (step 3). The most controversial issues relate to the following:

(1) The type and scope of IP protection of the creations and innovations related to these stages of the 3DP process;
(2) The application of some exceptions and limitations to the rights;
(3) The assessment of IPR infringement liabilities [23]

Concerning (1) protection, the most controversial issues relate to the IPR protection of the CAD file and the design data. Regardless of whether the CAD file is created from scratch or whether it is developed via 3D scanning a pre-existing object, it is unquestionable that such a file, as well as the related technical data, represent the most valuable elements in the 3DP process, making protection desirable [23].

From the point of view of a clinician or designer working with medical 3DP, IP rights may not seem like the most pressing issue. However, even if seeking protection for the CAD files developed might not be a priority, a designer would be interested in ensuring that third parties’ IP rights are not infringed during her/his activities. Issues of protection and infringement are interlinked—if protection of CAD files is not possible via IPRs, for instance, then it is also unlikely that third parties’ infringement will arise.

To date, there are several challenges in terms of whether and to what extent IPR can apply to CAD files and design data. For instance, a 3D model can potentially be considered a work of art per se, independent from the physical artifact it represents, and thus, can attract copyright protection by itself. For instance, in Cofemel [24], it was clarified that no other requirement than sufficient originality of the design at stake is needed for copyright protection to arise.

However, although a CAD file might be considered a ‘literary work’, more specifically, a computer program under copyright law, a definition to this effect does not currently exist in EU copyright law [23]. In the domain of patent law, it is unclear whether patents can be used to protect CAD files. For example, it might be challenging to determine when a technical effect and technical contribution are present [25]. Although new strategies to file ‘CAD-files’ types of patent claims are on the rise, whether patent offices will accept them remains an open question. In terms of design rights, even though CAD files are not eligible for protection under the EU design rights framework, the design encompassed by a CAD file might be subject to design protection [26]. Finally, if a CAD file is downloadable, it can be protected as a service. In addition, the sign used to indicate the origin, thus distinctiveness of the CAD file from other files, might also be the object of trademark protection [23,27].

Generally speaking, IPRs do not protect (technical) data and information as such. As a consequence, the tools most often used to protect high-value technical data to date are trade secrecy and contractual agreements. Some IP tools, however, can provide limited protection to the creative or inventive elements linked to data. For instance, even if copyright law protects the expression of ideas, not ideas or facts as such, datasets could be protected via database rights [28]. Moreover, patent law might have some applications in protecting the technical inventions related to design data in the 3DP process [29].

IPR protection of CAD software is relatively uncontroversial, as the general rules of software copyright protection [30], patents for computer-implemented inventions (European Patent Convention, Article 52) [31], and trademarks for software-related classes apply. The regulatory framework for hardware (e.g., 3D printers and 3D scanners) is generally quite clear and developed, but there are some exceptions concerning the protection of processes, especially in relation to medical treatment, including diagnostic, surgical, and therapeutic methods [23]. In Europe, patents cannot be granted for medical processes if these methods are applied directly to the human body [32].

Concerning (2) exceptions and limitations to the rights, notably, IPRs are not absolute in the EU context, since they are subject to a set of established exceptions and limitations (E&L) which provide legitimate defenses for clinicians on possible infringement claims. E&L exist for patent [30], copyright [33], design (Community Design Regulation, Articles 20–23) [34], and trademark (European Union Trade Mark Regulation, Articles 14 and 15) [35].

The purpose of E&L is to ensure a balance between the interests of IP rights holders and users while protecting the good (in other words, between protection and access). Indeed, it is important to take E&L into account because in cases when an exception can apply, the presumed clinical infringer could be waived from infringement claims. 

In the EU, E&L vary to a large extent from country to country, and according to each IP right. However, in the context of the medical 3DP process from the perspective of clinicians, the most relevant exception is the research and experimental use (in patent law). The research and experimental use exception in the context of patent law allows performing tests and research for preparing regulatory approvals for a certain limited period before the end of a patent right. Even though much has been written concerning other exceptions like private and non-commercial use in the 3DP context (probably due to the strong maker community), the literature on the applicability of the research and experimental use exception is very scarce. This notwithstanding, it can be argued that the more 3DP technology will be used in healthcare, the more relevant this exception is likely to become. This is perhaps also one lesson that can be learned from the COVID-19 pandemic, where a rapid acceleration of the use of 3DP in healthcare took place—accompanied by IPR clashes [36,37].

Finally, in the context of (3) infringement, issues may arise at multiple stages of the medical 3DP process but might be especially relevant for the 3D modeling and the additive manufacturing stages. First, one should understand the difference between direct and indirect infringement. Direct infringement gives rise to primary liability and occurs when an unauthorized person performs certain forbidden activities (e.g., uses, makes, sells, imports, etc.) with an IPR-protected innovation within the territory of protection and during the term of protection. Indirect infringement gives rise to secondary liability and occurs when third parties without permission conduct certain activities (e.g., supply, offer to supply, etc.) not associated directly with the protected innovation itself, but that induce or otherwise aid the direct infringement. For instance, a clinician may be the direct infringer, and the hospital or device company may be the indirect infringer.

There is a large amount of detail that should be carefully considered in the context of IP infringement of medical 3DP, also because differences arise based on the types of IP rights at stake and of the jurisdiction [23]. Here, it suffices to point out that infringement might occur due to unauthorized acts realized in relation to (1) the CAD file, (2) the original object from where the CAD file was created (e.g., via scanning), as well as with (3) the software and (4) the hardware (e.g., use of the machines and the materials). It is therefore important for a clinician who utilizes any such items to make sure that no IPR are in place (which, as seen above, might not have a definite clear answer for CAD files), or, when there are, that appropriate licenses have been secured.

### 3.3. Data Protection and Privacy

Within the context of the European Union (EU), the importance of data protection is underlined by the EU Charter of Fundamental Rights, which provides the fundamental right of protection of personal data, and in Article 8 states that “data must be processed fairly for specified purposes and on the basis of the consent of the person concerned or some other legitimate basis laid down by law” [38].

From a practical perspective, the current and most relevant piece of legislation regarding data protection is the General Data Protection Regulation (GDPR) [39], which came into force on 25 May 2018 and is directly applicable in EU Member States. The GDPR regulates the processing of personal data of living persons (recital 27) by wholly or partly automated means (Article 2 (1)). The term “processing” is defined as “any operation or set of operations which is performed on personal data” and entails, inter alia, actions such as collection, recording, structuring, storage, adaptation or alteration, retrieval, and use of such data (Article 4 (2)). Personal data are defined as “any information relating to an identified or identifiable natural person” (Article 4 (1)). It suffices when the information directly or indirectly identifies the person.

Several phases of the medical 3DP process, such as imaging or 3D digitizing, fall under the scrutiny of the GDPR. In order to customize a device template for a particular patient the imaging and the CAD data based upon it will by necessity relate to a particular patient [40]. Therefore, as “information relating to an identified or identifiable natural person” the CAD data will relate to personal data in the meaning of Article 4 (1) and hence fall within its scope of application. Even where the data are pseudonymized by, for instance, exchanging the name of the patient with an identification code or a similar measure, the GDPR remains applicable as the data “could be attributed to a natural person by the use of additional information” [41] (Recital 26 (2)). Only anonymized data would fall outside of the GDPR’s scope of application.

The phases of the 3DP process also fall within the wide scope of the definition of ‘processing’ in Article 4 (2). This definition would also encompass the distribution of data between different entities involved in the production process, such as when the 3D modeling process is conducted by a different entity than the one producing the medical imaging of the patient. Additionally, if the patient’s data are transferred to an entity outside the EU, e.g., a 3D printing facility abroad, it needs to be assured that the country provides an adequate level of data protection [40].

The GDPR also restricts the extent to which data may be used through, *inter alia*, the principles of “purpose limitation” and “data minimization”. The former means that the collected data may only be used for the specified and explicit purposes and may not be further processed in a manner ‘incompatible’ with such purpose, while the latter stipulates that the processing must be “adequate and limited to what is necessary in relation to the intended purposes”. Both principles may limit the further use of patient data. Finally, the GDPR also mandates that the data ought to be destroyed once their storage is no longer necessary for the initial purpose or when the data subject withdraws consent.

The importance of protecting personal data within the GDPR is underlined by Article 6 which provides a general prohibition of processing of personal data. This means that processing such data is only lawful in exceptional cases. One of these exceptions applies where the data subject has provided consent (Article 6 (1)) and will be the usual form for data controllers to comply with the GDPR. Such consent is defined as “freely given, specific, informed and unambiguous indication of the data subject’s wishes by which he or she, by a statement or by a clear affirmative action, signifies agreement to the processing of personal data relating to him or her.” [39,40]. It must be given through an affirmative act and should cover all activities relating to the processing of data for the same purpose or purposes [42] (p. 589). This means that such consent would usually need to be obtained before conducting the medical imaging and the other subsequent steps of processing.

Among personal data, biometric and health data (as well as some other data) are offered special protections. Biometric data results “from specific technical processing relating to the physical, physiological or behavioral characteristics of a natural person, which allow or confirm the unique identification of that natural person […] such as facial images” [39]. At least CT scans of facial bones should fall under this definition since they can be processed to yield facial phantoms that can fool facial recognition software [43], but, arguably, this could extend to several other forms of imaging-based data as well. Health data encompasses information concerning the “physical or mental health status of the data subject” [39], and could therefore also apply to many forms of data based on imaging.

Qualification of 3D models as biometric or health data would have far-reaching consequences as it would make the processing of data more burdensome since the data controller would then need to obtain the explicit consent of the data subject. This is a qualification in comparison to normal consent as discussed above. It means, for instance, that consent “cannot be implied, and requires a high degree of precision and definiteness in the declaration of consent, as well as a precise description of the purposes of processing.” [44]. Additionally, such explicit consent would also be required for further processing of the data in the medical 3DP process where such steps are not covered by the initial explicit consent.

However, explicit consent of the data subject is not required where the processing “is necessary for the purposes of preventive or occupational medicine, for the assessment of the working capacity of the employee, medical diagnosis, the provision of health or social care or treatment or the management of health or social care systems and services.” [39]. Relevant in this context would be the exception based on health care and provision which “covers all types of medical and social care, including diagnosis, treatment and prevention” [44] but must be limited to these purposes. The exception is subject to the rules within Article 9 (3) which prescribe that the data are processed under the responsibility “by or under the responsibility of a professional subject to the obligation of professional secrecy under [European] Union or [EU] Member State law or rules established by national competent bodies or by another person also subject to an obligation of secrecy under Union or Member State law or rules established by national competent bodies”. Such professional secrecy relates to doctors, dentists, and hospitals. Since professional secrecy is governed by the law of EU Member States, the conditions may differ from country to country [44].

## 4. Discussion

In this study, using legal doctrinal study and legal informatics, we analyzed relevant EU legislation and case law in four legal areas previously identified as relevant to medical 3D printing. For pre-market approval, most patient-matched medical devices were found to follow the standard regulatory pathway, with the role of the custom device exemption being somewhat uncertain. For post-market liability, the regulatory framework was found to be in flux, with the current framework somewhat poorly equipped to handle liability in the age of digital products and distributed production chains. The major uncertainty identified regarding IPR was the status of protection offered to CAD files created or used in the 3DP process. For data protection purposes, the CAD files were found to be subject to the GDPR and probably to come under the increased protection requirements of biometric or health data. Some of the issues found will be addressed more closely in the following paragraphs.

While there have been previous papers concerning specific legal issues relevant to 3DP in the EU, this study is to our knowledge the first to comprehensively examine the legal challenges surrounding the technology and its medical usage as a whole. Strengths of this study include the multidisciplinary nature of the study team, which allowed us to formulate clinically relevant questions regarding legislation, and the broad range of sources (primary legislation, case law, and scholarly legal study) used to address the study questions. On the other hand, the broad scope of this study necessitated limiting the depth of study afforded to each question. In many cases, we were unable to provide definite answers to the questions identified, which is based in actual uncertainties in the regulation concerned. Presenting the findings of this study as a research paper has been an additional challenge because of the interdisciplinary nature of the study and the differing approaches to academic reporting between the legal, technical, and medical fields.

### 4.1. Special Questions

#### 4.1.1. Crisis Response

Besides the two main advantages of complex internal/surface structure and mass customization, a third property of 3DP is the flexibility of production capacity to quickly react to unexpected demand. This was well demonstrated in the recent COVID-19 pandemic and remains an important consideration for future crises. In the initial phases of the pandemic, 3DP was used to remedy the surge of demand and acute disruption of supply chains for several medical supplies and devices. Examples included ventilator parts, nasal swabs for taking test samples, face shield holders, and personal protective equipment [45,46]. Most of these products are regulated medical devices and would normally require certification and approval. Some countries speeded up the regulatory process or approved temporary exceptions to be able to utilize these, and in other countries, the need was so high that regulations were bypassed. In this setting, all products were of a mass-produced type, with 3DP used to quickly recruit production capacity while traditional supply chains were disturbed, or production was being re-tooled. The legal challenges presented are quite different than those found in other medical 3DP and are dealt with more closely by [47,48].

#### 4.1.2. Bioprinting and Pharmacoprinting

Besides traditional (mass-produced or personalized) medical devices, 3DP also brings along the possibility of *bioprinting* and *pharmacoprinting*. Bioprinting may be defined as 3DP using materials containing viable cells, and once the underlying biology is mastered, it is expected to produce artificial tissues and even organs. At the moment, however, while research usage is relatively widespread, clinical applications are still some way off. Cell therapies are already in use and regulated, and regulatory schemes for bioprinting have aspects of both medical device and cell therapy regulations.

Pharmacoprinting means 3DP using pharmaceuticals as material, to produce either oral dosage forms [49] or other dosage forms such as implants [50]. Pharmacoprinted products would typically be regulated either as drugs or as combination/advanced therapies.

For the purposes of this article, we decided not to cover questions unique to bioprinting or pharmacoprinting in greater detail.

### 4.2. Hospitals as Manufacturers 

The health institution exception of the MDR allows for disregarding certain requirements when an MDR is not placed “on the market” but instead produced and used within a healthcare facility. Certain extra qualifications to this rule include that the production shall not happen “on an industrial scale”, the devices “are not transferred to another legal entity”, and no equivalent device existing on the market can fulfill the needs of the patient “at the appropriate level of performance”.

This leaves open several questions. Can the lead time of production be considered when determining “the appropriate level of performance”? What about the situation where an industrial partner is co-located at the hospital to provide 3DP services as has been suggested—does this different legal entity preclude utilization of the exemption? Does it mean that the whole production process must happen within the legal entity of the hospital, or can any outside consultants or production services be utilized? Finally, what about the projected future developments where hospitals will buy IP and produce their own physical devices? The physical manufacturing would in this case happen at the health institution and would not necessarily be “at an industrial scale”. On the other hand, if the exemption would not apply and such devices would be subject to the full requirements of the regulations, what would such a business model require in terms of production chain standardization, and how would the liability be distributed?

### 4.3. Custom Devices versus Patient-Matched Devices

There has previously been some speculation over whether the MDR custom device exemption would apply to most 3DP devices. At the same time, the meaning of “custom device” has been somewhat vague, with the FDA differentiating between “custom” and “customized” devices. Acknowledging the terminological confusion, the International Medical Devices Regulators Forum (IMDRF), which attempts global harmonization of medical device regulations, has published a document regarding definitions for personalized medical devices [51]. Under this nomenclature, “personalized medical device” is an umbrella term, encompassing ‘custom’ or ‘custom-made’ devices, which are produced “in accordance with a written request by an authorized professional...under their responsibility”, ‘patient-matched’ devices are “designed and produced under the responsibility of the manufacturer”, and ‘adaptable’ devices are assembled to suite the patient’s needs at the point of care.

The MDR custom device exemption seems to coincide with the definition of ‘custom-made’ devices, but little specific consideration is given to “patient-matched” devices. It is, however, in this “mass-customization” scenario where currently available medical 3DP can arguably have its greatest clinical impact, if, for example, it becomes routine to use a patient-specific implant for a certain kind of surgery, as is, for instance, reported to have happened in orbital floor fractures at some facilities [52]. It is unlikely that every new implant presents a unique case, and therefore stronger regulatory protections than required for a “custom device” could be asked for. FDA requirements for devices to qualify as custom as opposed to customized are quite explicit (e.g., only five devices of the same class are produced per year per manufacturer). EU regulations, in comparison, appear to be vague on this point.

A newer Q and A document attempts to align the MDR and IMDRF classifications [7]. This document explicitly addresses 3DP and states that it does not automatically qualify a device for the custom device exemption, and that ”patient-matched medical devices (as defined by IMDRF) are not qualified as CMDs and must follow the ‘standard’ MDR regulatory pathway for placing on the market”. It also states that “industrial manufacturing processes...such as CAD CAM, 3D-printing” may be used to manufacture CMDs, as long as they are not “mass-produced”. The definition of “mass production” is still left open, unlike in FDA regulations where a clear limit (5 devices of a certain type per year) is provided. Also, whereas the FDA Emergency Authorization access to uncertified devices requires demonstration that alternative devices on the market cannot satisfy the requirements, the EU CMD exemptions do not seem to restrict access to custom devices even after a CE-approved product exists on the market.

Given that the guidance makes it clear that patient-matched devices are supposed to be regulated by normal pathways, the fact that they are given no specific consideration in the MDR seems like an omission. Patient-matched devices come with their specific regulatory concerns (for instance, the accuracy of the imaging and software toolchain). 

### 4.4. Medical Models

The legal role of 3D printed anatomical models, at least initially the largest group of medical 3D products, and a seemingly low-barrier entry into on-site medical 3DP, has been a contested topic for some time.

Even though its scope is limited to regulating products on the market, the FDA has been proactive in discussing whether medical models are considered medical devices in the US. Earlier, their view seemed to be that if the model can affect the diagnosis, management, or treatment of a specific patient then it is considered a Class II medical device. Examples of non-regulated models (i.e., not medical devices) would be models meant for teaching, visualizing a pathology in general, but not for a specific patient, or communicating findings to a patient, in which case they may be patient specific.

Despite this, currently, the FDA has not classified 3DP medical models manufactured at the point of care as medical devices themselves (and indeed the FDA would lack the jurisdiction to enforce such a classification). However, 3DP models marketed for diagnostic use should be prepared by a toolchain (software and printer) that has been cleared by the FDA for such an indication. For point-of-care production of such models, using an FDA-approved toolchain is just a “best practice” recommendation [53].

The proposed legislation in Australia [54] shares many similarities, where models are considered medical devices, and individual clinicians producing them would have to assume the full responsibility of a manufacturer. The Australian Therapeutic Goods Administration also publishes proactive guidelines on medical device classification under the coming new legislation, including the distinction between patient-matched and custom-made medical devices [55]. The legislation also aims to introduce a new concept of a Medical Device Production System (MDPS). This would allow, in the context of medical models, manufacturers to place on the market toolchains containing the software and printer that would enable clinicians to produce medical models without having to worry about specific compliance issues or taking on the responsibility of a manufacturer.

No EU instance has shown similar initiative or released equivalent authoritative views or guidelines, but in personal communications representatives of the EMA have stated that whether 3D printed models of the patient’s anatomy are considered medical devices “will depend on the intended use and whether this meets the definition of a medical device as per Regulation (EU) 2017/745 (MDR), Article 2.”. Since the definition of a medical device under the MDR is “any instrument […] for diagnosis, prevention, monitoring, prediction, prognosis, treatment or alleviation of disease […] injury or disability, investigation, replacement or modification of the anatomy or of a physiological or pathological process or state”, the definition would appear to be quite analogous to the one under the FDA. However, the EU MDR does have the jurisdiction to regulate point-of-care production (the Health Institution Exemption).

The legal status of 3DP medical models in the EU is therefore still hanging in the air, but, for the time being, it would seem that with no MDPS-like innovations on the horizon, manufacturing medical models (and indeed any point-of-care printing) in hospitals, even if using printers and software meant for that purpose, would still require full compliance with the production requirements (Health Institution Exemption applying), e.g., quality control system, etc. This is a heavy requirement and puts EU-based health institution actors in a markedly disadvantaged position compared to US or Australian colleagues.

Under the MDR, it is also unclear whether parts of the toolchain used to produce a custom device would have to be approved. In a guidance document, it is stated that using CE-marked devices in the toolchain may help in proving that regulatory requirements are fulfilled, but the user/manufacturer of the custom medical device is still solely responsible for its compliance and approval [7].

### 4.5. Are We Being Overcautious?

3DP is widely regarded as a ‘disruptive’ technology that makes it possible for complex products to be fabricated more easily. Democratization of production means that what previously required multi-disciplinary knowledge, large corporate budgets, and expensive means of production can now be achieved using a machine located in someone’s garage, with software downloaded over the Internet, for little more than the cost of a standard computer. It is already quite feasible to print medical models on hobbyist 3D printers, and more advanced forms of production (such as stereolithography and metallic 3DP) are also being made available to consumers.

It has already been argued that the real benefit from the ‘democratization’ of design and manufacturing technology enabled by medical 3DP may lie outside of Europe, more specifically in parts of Asia, Africa, and South America [56]. These are to some extent regions where the scarcity, quality, and cost of medical treatment means that many people suffer from problems that do not even occur in Europe.

While this may be true, increasing evidence suggests that 3DP and associated technologies can provide better quality of treatment even in high-income health systems. The gap between technical capability and practical feasibility in Europe is, however, wide. Custom-made or patient-matched medical devices seem to be relegated to the domain of relatively large companies able to navigate and afford the bureaucratic overhead, with smaller start-ups or hospital departments, both technically capable and in the latter case very much already in the business of ensuring optimal outcomes for patients, having to stay outside the playing field unless considering fringe, last-ditch efforts. 

While the concept of personalized medicine may be seen as novel, surgical care has in some ways always been personalized, as no two operations are exactly the same. As an example, the current paradigm for personalizing orthopedic care relies on the surgeon using modular prosthetics or kits of screws, plates, etc., to design a working solution in real-time, sometimes modifying plates e.g., by bending, while the patient is anesthetized and cut open. When this bricolage fails or produces poor results the surgeon may be blamed, but the individual pieces of material are rarely seen as defective. It could well be argued that performing this creative process in silico, i.e., planning the operation and producing a customized implant or guides, given the possibilities of infinite undo, advanced modeling capabilities, and shortened operative time with associated decreased morbidity, should not be artificially made more cumbersome by legislation than the current standard of care. 

The driving force behind all decisions regarding relevant legislation should in the end be producing the best possible outcomes for the largest number of patients. A non-existent intervention is bound to have zero complications but also to have zero positive health impact. Optimal medical device safety may therefore not equal optimal patient outcomes.

For these reasons, it will be more important than ever to carefully think about more adaptive legal and regulatory frameworks for such technologies. One example of such approaches can be found in the forthcoming EU AI act [57] and its rules on so-called “regulatory sandboxes” in Art. 53.

## 5. Conclusions

The EU MDR, though much more extensive in its requirements than the previous medical device directive, seems to have missed the opportunity to address key contemporary and future developments of modern medicine, including personalized medical devices, medical models, and point-of-care production, leaving it outdated at its introduction. There is much uncertainty regarding the liability regime and the intellectual property rights relevant to medical 3D printing in the EU, and the General Data Protection Regulation, as a very wide and restrictive regulation, also places several requirements on the medical 3DP process. The sum of these factors is that the medical 3DP landscape in the EU does not seem very welcoming to innovation.

When considering technical innovation, there is a tendency of the EU legislature and judiciary to follow a ‘cautious’ approach when balancing the interests of the parties involved [58], thus minimizing both potential risks and rewards.

One cannot stop wondering whether a ‘better’, perhaps less conservative, legislative framework could foster developments and expand the range of uses of medical 3DP. It is understood that patients, as being in a disadvantaged situation, require special legal protection. However, it should also be recognized that even in advanced and well-funded health systems there is a big unfulfilled need for new and better therapies where the current capabilities of medicine are not able to provide sufficient help. This very real suffering and even death should also be kept in mind when considering how strictly to regulate medical innovation.

Medical innovation, however, will continue both within and (especially) outside the EU, and new methods tend to be rapidly adopted worldwide where law permits. In cases where it does not, the law may need to adapt under pressure from stakeholders such as patients, clinicians, and device manufacturers.

## Figures and Tables

**Table 1:** Regulatory focus.

Regulation Phase	Pre-Market	Post-Market
Regulatory focus	Safety and efficacy	Traceability and liability
Key issues	Medical device regulationclassification	Surveillance and monitoringProduct liability and Tort liability
	Market approval pathway	Contracts and consumer protection
	Medical Device	The definition of product defects in digital files
	Software as a medical device	The demarcation of liability

**Table 2:** IPR types, subject matter, and purpose.

Type	Subject Matter and Purpose
Copyright	Right related to original/creative works, including literary, theatric, musical, and other artistic works (including software); right is only against copying and lies in the expression of an idea rather than its general concept or character. In the EU, copyright attaches automatically to the creation with no need for registration and lasts for 70 years after the death of the author.
Patents	A negative exclusionary right that can be obtained for technical inventions that are novel, involve an inventive step, are industrially applicable, and sufficiently disclosed. Patentholders are allowed to exclude others from practicing the invention in exchange for public disclosure of the invention (quid pro quo). Patents are granted after a formal examination process, and they generally last for 20 years after the filing date.
Trademark	Right to exclusive use of any sign (e.g., words, letters, numerals, pictures, shapes, colors, sounds, smells, etc.) by which consumers can identify the source of goods or services. In the EU, trademarks should be registered through a formal examination process; they can last indefinitely if they are renewed.
Industrial designs	Right to the original, ornamental, and non-functional feature (i.e., the appearance) of the whole or part of an industrial or handcrafted product resulting from the features in the lines, contours, colors, shape, texture, and/or materials used. In the EU, designs can be registered through a formal examination process (registered design) or arise automatically without registration (unregistered design). They last up to 25 years.

## Data Availability

Data are contained within the article.
